# Functional Outcomes in Patients With Prostate Cancer Undergoing Frozen Section Guided Radical Prostatectomy

**DOI:** 10.1111/iju.70115

**Published:** 2025-05-20

**Authors:** Moritz J. Reike, Karl H. Tully, Maximilian Schulmeier, Alina Reicherz, Henning Bahlburg, Guido Müller, Peter Bach, Carlo Sternemann, Andrea Tannapfel, Joachim Noldus, Marko Brock, Florian Roghmann

**Affiliations:** ^1^ Department of Urology Marien Hospital Herne, Ruhr‐University Bochum Bochum Germany; ^2^ Institute of Pathology, Ruhr‐University Bochum Bochum Germany; ^3^ Department of Urology Stiftungsklinikum PROSELIS Recklinghausen Germany

**Keywords:** continence, frozen section, potency, prostate cancer, radical prostatectomy

## Abstract

**Purpose:**

Patients with an intermediate risk prostate cancer (PCa) are not routinely offered nerve sparing (NS). Implementation of whole surface frozen section (FS) made NS‐radical prostatectomy (RP) available and oncologically safe. In the present study, we aimed to assess the impact of the addition of FS on NS during RP and potentially improved functional outcomes.

**Methods:**

Institutional data of patients (PSA ≤ 20 ng/mL, Gleason‐Score ≤ 7, cT1c‐cT2c) undergoing RP between 06/2011 and 11/2014 were prospectively collected. Decision for NS was made by the surgeon supported by FS. Only patients with a preoperative International Index of Erectile Function (IIEF‐5) ≥ 17 were analyzed for potency. Continence was defined as pad use of ≤ 1 pad/day. Separate multivariable regression analyses were employed to examine predictors for both endpoints (i.e., potency and continence).

**Results:**

Overall, 702 patients were included in this study. Final nerve‐sparing surgery was performed in 671 patients (95.6%). Before the introduction of FS, only 392 patients (56%) would have undergone NS‐RP. FS enabled intraoperative/final NS for 688 (98%) and 666 (95%) patients. No differences at overall follow‐up between low‐ and intermediate‐risk patients regarding continence (*n* = 122 (84.1%) vs. *n* = 223 (82.6%), *p* = 0.689) and potency (IIEF‐5 ≥ 17, *n* = 16/47 vs. 26/68, *p* = 0.646) were detected. Surgeon volume was associated with improved continence (OR 3.69, 95% CI 1.86–7.32, *p* < 0.001) and erectile function (EF) (OR 2.49, 95% CI 1.23–5.03, *p* = 0.011).

**Conclusion:**

The introduction of FS expanded NS to patients with an intermediate‐risk PCa and selected high‐risk PCa. This may lead to improved functional outcomes as more patients were eligible for NS.

## 1 Introduction

Sparing of the neurovascular bundle (NVB) (nerve‐sparing, NS) during radical prostatectomy (RP) has been identified to be essential in preserving erectile function (EF) without negatively affecting oncological outcomes [1]. Additionally, NS is associated with improved urinary continence after RP, and can be seen both as a surrogate for a meticulous procedure and careful apical dissection of the prostate [2, 3]. Current guidelines emphasize the application of NS in appropriate patients [4]. However, neither a clear statement is made on exact clinical preconditions (i.e., low‐risk patients) nor the usage of different levels of NS. Surgical strategies and algorithms allowing oncologically safe NS procedures rely on imaging and quantitative biopsy results, which seem to be inadequate to ensure the best possible rate of NS procedures.

Therefore, we currently rely on frozen section (FS) during RP as the only strategy to combine the best possible functional outcome without risking unfavorable oncological results [3, 5, 6, 7]. As previously described in greater detail, FS has been shown to reduce positive surgical margins (PSMs) while allowing the surgeon to maximize NS [7]. Until the introduction of this technique in 2011, bilateral NS had only been offered to patients with low‐risk disease (according to D'Amico criteria) [8]. On the other hand, unilateral NS was offered to patients with a single‐sided Gleason score of 4 and/or palpable tumor (≤ cT2b). The rationale for surgical approaches before FS had been deduced from studies that described risk parameters for PSMs after RP [9, 10]. Nowadays, a considerable portion of low‐risk patients choose active surveillance, resulting in a changed clientele for NS RP [11].

Oncologic safety of NS in the intermediate‐risk prostate cancer (PCa) cohort has been demonstrated in the present cohort by Tully et al. [5].

The purpose of the present study was to evaluate the rate of NS during surgery and to examine the effects of NS on functional outcomes in a patient population of intermediate‐risk PCa and selected high‐risk patients (PSA ≤ 20 ng/mL, Gleason‐Score ≤ 7, cT1c‐cT2c) that underwent FS‐guided RP.

## 2 Patients and Methods

### 2.1 Data Source and Study Population

Data was obtained from a prospectively collected institutional database of men with histologically confirmed PCa (PSA ≤ 20 ng/mL, Gleason score ≤ 7, cT1c‐cT2c) undergoing ORP or RARP between 06/2011 and 11/2014. Patients were stratified according to D'Amico et al. [8].

All patients received a whole surface FS during RP, as described in the literature [7]. After removal of the prostate, the specimen will be removed from the peritoneal space during the operation. The pathological examination is described in detail in the literature [7]. Results from the FS are usually available after 45 min. Dedicated genitourinary pathologists performed all pathological analyses of FS and the final pathological review. In the case of PSM, the respective locations were reported to the surgeon using a standardized template (Figure S1). Secondary tumor resection in the corresponding section was then performed accordingly [8]. The extent of a second resection after FS was decided by each surgeon with no institutional guideline. Therefore, it is possible that even with a positive FS, a sufficient NS has been performed. NS was defined by the surgeon as unilateral or bilateral preservation of the NVB.

**FIGURE 1:**
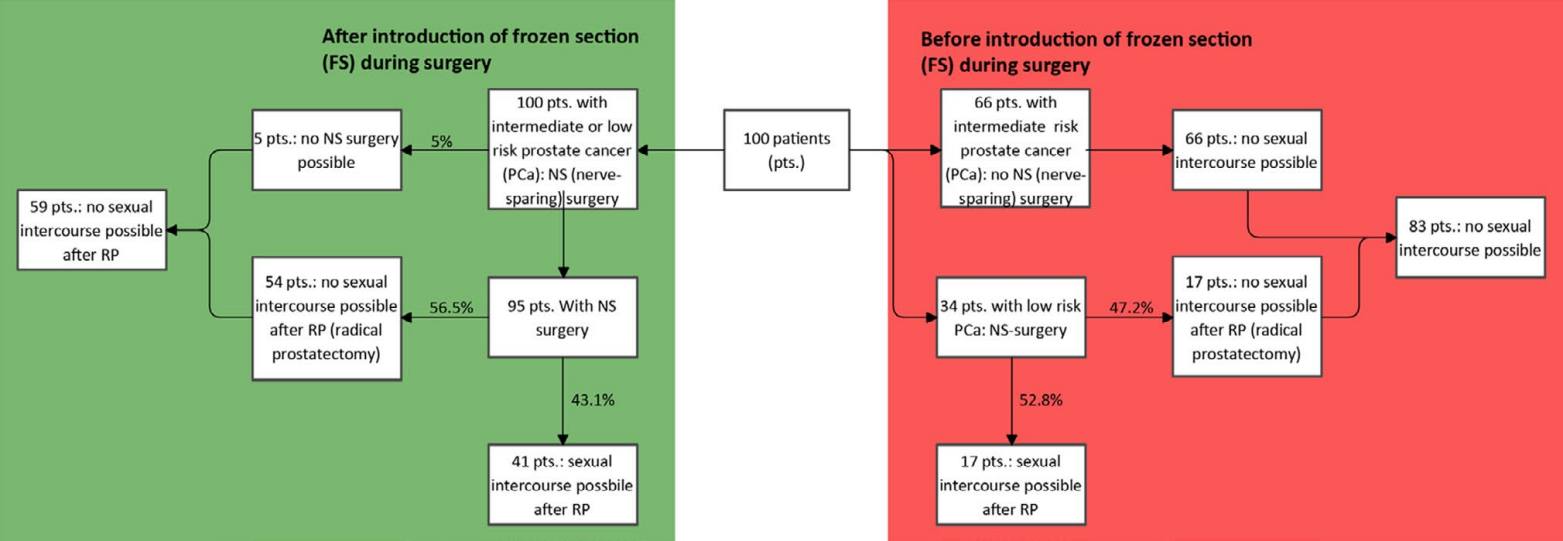
Flowchart displaying the extrapolated postoperative erectile function of 100 hypothetical patients that underwent radical prostatectomy (RP) with low/intermediate risk as well as selected high risk prostate cancer patients (PSA ≤ 20 ng/mL, Gleason score ≤ 7, cT1c‐cT2) according to the present functional results before (shown in red) and after the introduction (shown in green) of frozen section (FS) into daily clinical practice. Introduction of FS improved the likelihood of possible sexual intercourse after RP in the whole cohort by 1.7‐fold.

Figure 1 depicts decision algorithms for NS before and after the introduction of FS in 100 hypothetical patients. The data sets generated during and/or analyzed during the current study are available from the corresponding author on reasonable request.

### 2.2 Outcome Measurement

The main goal of the present study was to investigate predictors of NS during RP and to examine functional outcomes after RP with intraoperative FS. For functional outcomes, we defined two surrogate endpoints:

1. Potency, measured using the “International Index of Erectile Function” (IIEF‐5) score (patient questionnaire to validate EF; a IIEF score ≥ 17 is classified a mild erectile dysfunction [ED], ≥ 22 signals no ED) [12]. The patients filled out the IIEF‐questionnaire on the day of admission before surgery.
2. Continence, defined as ≤ 1 pad/day.

### 2.3 Covariables

Preoperative baseline characteristics for all patients included age at the time of surgery, preoperative PSA (ng/mL), prostate volume (ccm), clinical T‐stage as defined by the Union for International Cancer Control (UICC), and Gleason score at biopsy as described by Epstein et al. [13]. The surgical approach (ORP vs. RARP) and surgeon volume (≥ 100 RP/year vs. < 100RP/year) as well as pathological characteristics such as tumor volume, final T‐stage, and final Gleason score were evaluated.

### 2.4 Statistical Analysis

Medians and interquartile ranges (IQRs), as well as frequencies and proportions, were generated for continuous and categorical variables, respectively. We employed the Mann–Whitney *U* test to examine differences in continuous variables and Pearson's chi‐square test to examine differences in categorical variables. Ensuing, multivariable logistic regression models examining the preoperative and postoperative covariables mentioned above were employed to examine the association with intraoperative NS and postoperative functional outcomes, respectively. The functional outcomes of the present cohort were extrapolated to 100 hypothetical patients that underwent the current RP approach via FS‐guided NS in comparison to the former approach with preoperative NS decision, according only to feasible in patients with low‐risk tumor characteristics.

All analyses were performed using Stata (Version Stata/SE 15.1, Stata Corp LLC, Texas, USA). The institutional ethics committee approved this study (Approval number 2015‐5250).

## 3 Results

### 3.1 Baseline Characteristics

In total, 702 consecutive patients who underwent RP were included in our cohort. Table 1 gives a detailed overview of our study population.

**TABLE 1:** Baseline and tumor‐specific characteristics.

	Overall *N* = 702	Low‐risk *N* = 237 (33.8%)	Intermediate‐risk/high‐risk *N* = 465 (66.2%)	*p*
Median age, years (IQR)	64.6 (59.9, 69.7)	63.4 (58.8, 68.2)	65.2 (60.6, 70.4)	0.001
Median preoperative PSA, ng/mL (IQR)	6.6 (5.1, 9.4)	5.8 (4.8, 7.4)	7.2 (5.4, 10.6)	< 0.001
Median prostate volume, ccm (IQR)	37 (30, 48)	40 (40, 50)	37 (29, 48)	0.049
Median number of positive biopsies, *n* (IQR)	3 (2, 5)	2 (1, 4)	4 (2, 6)	< 0.001
Clinical T‐stage at DRE, *n* (%)				
cT1	471 (67.1)	237 (100.0)	234 (50.3)	< 0.001
cT2	231 (32.9)	0 (0.0)	231 (49.7)	
Tumor laterality, *n* (%)				
Right	199 (28.4)	69 (29.1)	130 (28.0)	0.314
Left	191 (27.2)	73 (30.8)	118 (25.4)	
Both	307 (43.7)	94 (39.7)	213 (45.8)	
Unknown	5 (0.7)	1 (0.4)	4 (0.9)	
Gleason‐score, *n* (%)				
6	395 (56.3)	237 (100.0)	158 (34.0)	< 0.001
7a	223 (31.8)	0 (0.0)	223 (48.0)	
7b	84 (12.0)	0 (0.0)	84 (18.1)	
Surgical approach, *n* (%)				
Open	479 (68.2)	157 (66.2)	322 (69.3)	0.419
Robotic	223 (31.8)	80 (33.8)	143 (30.8)	
Nerve‐sparing				
None	31 (4.4)	6 (2.5)	25 (5.4)	< 0.001
Unilateral	178 (25.4)	30 (12.7)	148 (31.8)	
Bilateral	493 (70.2)	201 (84.8)	292 (62.8)	

### 3.2 Nerve Sparing Surgery

For all patients, unilateral or bilateral NS was attempted and the majority of patients underwent final unilateral (*n* = 178/702, 25.4%) or bilateral (*n* = 493/702, 70.2%) NS, while 31 patients (4.4%) of our cohort ultimately underwent surgery without NS. Nine patients received surgery without the attempt of NS and five patients had an unknown tumor laterality but received NS.

Final uni‐ or bilateral NS was performed in 97.5% (*n* = 231/237) of patients with a low‐risk PCa, whereas in intermediate‐risk/selected high‐risk patients, 94.6% (440/465) received NS. As shown in Table 2, NS without the availability of FS only feasible in patients with low‐risk and/or a single‐sided Gleason score of 4 and/or palpable tumor (≤ cT2b) would hypothetically have been performed in only 392/688 (57%) patients. Availability of intraoperative FS increased intraoperative NS attempts by the surgeon to all patients and led to final NS in 666/688 (96.8%) of patients.

**TABLE 2:** Comparison of nerve‐sparing (NS) surgery without and with guidance of FS.

	NS before the introduction of FS *N* = 392/688 (57%)	Intraoperative NS with support of FS, *N* = 688/688 (100%)	Final NS with support of FS, *N* = 666/688 (96.8%)
Tumor laterality, *n* (patients (pts.) with NS/total pts.)			
Right	115/198	198/198	197/198
Left	117/190	190/190	181/190
Bilateral	160/300	300/300	288/300
	9 pts. received surgery without NS 5 pts. with unknown tumor laterality, but received NS		

On multivariable logistic regression (Table S1), a high proportion of positive biopsies (> 75%), or a higher Gleason score (≥ 7) was associated with lower odds of NS (proportion of positive biopsies > 75%: odds ratio (OR) 0.28, 95% CI 0.08–1, *p* = 0.05; Gleason score ≥ 7b: OR 0.39, 95% CI 0.15–1.01, *p* = 0.05). Moreover, high‐volume surgeons were more likely to perform surgery with NS (OR 2.49, 95% CI 1.04–5.93; *p* = 0.04).

### 3.3 Continence

One year follow‐up was available for 485/702 (68.8%) patients. The median follow‐up was 31 months (IQR 23–41). After RP, 402/485 (82.9%) and 345/415 (83.1%) patients were continent at the 1‐year and final follow‐up, respectively (Table S2).

At 1‐year follow up, 27/170 (15.9%) patients with unilateral and 56/315 (17.8%) patients with bilateral NS were classified as incontinent. Comparison of low‐risk and intermediate‐risk patients showed no significant difference regarding their outcome. After complete follow‐up, 122/145 (84.1%) patients in the low‐risk group and 223/270 (82.6%) in the intermediate‐/high risk group reported continence. Again, no significant difference regarding continence was detected.

Continence was not significantly influenced by surgical approach, age, or prostate volume (*p* > 0.05). However, the surgical volume of the surgeon was a significant predictor of urinary continence (OR 3.85, 95% CI 2.19–6.75, *p* < 0.001, Table S3). NS (unilateral or bilateral) was not associated with increased chances for continence at 1‐year follow‐up (OR 1.13, 95% CI 0.36–3.55, *p* = 0.831 and OR 1.33, 95% CI 0.43–4.08, *p* = 0.621, respectively, Table S3).

### 3.4 Potency

Complete preoperative and postoperative IIEF‐5 scores were available for 137/702 (19.5%) patients without prior ED (median final follow‐up 33 months (IQR 25–42)). At 1‐year follow‐up, 38/137 (27.7%) patients indicated an IIEF‐5 score ≥ 17. Data about EF sufficient for sexual intercourse was available for 448/702 (63.8%) patients without ED presurgery, with a median final follow‐up of 33 months (IQR 25–42).

For 65.4% (*n* = 293/448) of patients, EF was insufficient for sexual intercourse 1 year after RP. Regarding the overall follow‐up, 174/404 patients (43.1%) were able to perform sexual intercourse. Patients with a bilateral NS procedure reported higher rates of successful intercourse compared to unilateral NS (46.6% vs. 33.6%, *p* = 0.0122, Table S4).

Prostate volume, a high preoperative PSA, and detectable tumor via DRE did not lower the odds of sufficient EF (Table S4). High surgeon volume was associated with increased odds of EF after RP (OR 2.18, 95% CI 1.24–4.83, *p* = 0.006, Table S5). NS (unilateral or bilateral) was not associated with increased chances for sufficient EF at 1‐year follow‐up (OR 1.12, 95% CI 0.30–4.22, *p* = 0.865 and OR 1.73, 95% CI 0.47–6.36, *p* = 0.407, respectively, Table S5).

Extrapolation of the present functional outcomes to 100 hypothetical patients that underwent either FS guided NS‐RP or RP with NS decision according to preoperative risk assessment suggests a 1.7‐fold absolute increase in potency (i.e., possible sexual intercourse); 42 patients (with FS) versus 25 patients (without FS) by the introduction of routine FS (Figure 1).

## 4 Discussion

In our cohort, the introduction of FS expanded the possibility of NS to selected high‐risk and all intermediate‐risk patients. This expansion may lead to improved functional outcomes in absolute numbers as more patients were eligible for NS. Based on the present functional outcomes, we estimated that FS improved the likelihood of possible sexual intercourse after RP by 1.7‐fold in 100 hypothetical patients.

Protection of nerves, vessels, and connective tissue surrounding the prostate as well as the bladder neck, and functional urethra during RP improves postoperative functional results [1, 3, 9]. Especially, preservation of the NVBs improves the chances of sufficient EF after RP and is associated with higher levels of continence after RP in the first 6 months [14, 15].

One main concern in performing NS is its oncological safety. In our cohort, the safety of NS is supported by whole mount FS, which allows the surgeon to attempt a maximal NS and modify the procedure by further resections after FS to secure its oncologic safety. We demonstrated and discussed the oncologic safety of FS and the approach to NS in the present cohort before [5]. Prior to introducing FS, bilateral NS was only performed in low‐risk patients and unilateral NS in patients with a contralateral single‐sided Gleason‐score of 4 or nonpalpable tumor [7, 9, 10]. Due to intraoperative whole surface FS, NS is now offered to a higher number of patients with localized PCa. Without FS, uni‐r bilateral NS would not have been performed in 43% of patients, who now had the chance of NS during RP. After FS, NS was possible in 96.8% of patients. These results confirm outcomes by Schlomm et al., who increased the rate of NS by FS as well [6]. Based on our results we calculated a 1.7‐fold increase of the hypothetical probability of possible sexual intercourse in our cohort (Figure 1), which underlines the benefit of performing RP with whole mount FS.

As expected, the chances of bilateral NS were significantly reduced in more advanced PCa stages, which is based on the higher probability of nerve‐infiltrating tumor growth [1]. Confirming previous studies [16, 17], no statistically significant difference regarding the odds of bilateral nerve‐sparing based on the surgical approach was shown.

Reeves et al. reported a significant difference in continence up to 6 months after surgery by the addition of NS (NS 88.9% vs. non‐NS 69.8%). However, long‐term continence after RP was not significantly improved by NS [15]. In contrast, and in line with our results, Michl et al. [3] reported a significant improvement in long‐term continence in patients with NS compared to non‐NS techniques (85.4% vs. 70.5%). The authors suggested that these divergent findings are mainly based on significant bias in the baseline characteristics of the selected patients by Reeves et al. Avulova et al. reported similar effects of nerve‐sparing surgery [16]. Following the arguments by Michl et al. [3], the introduction of FS and a higher number of NS attempts leads to an improved rate of bilateral NS and improved chances of continence after surgery. Therefore and based on our findings, we suggest offering NS to all patients with low‐risk or intermediate‐risk PCa and considering it in selected cases of high‐risk PCa. High surgeon volume not only decreases surgery‐associated morbidity and mortality, but in the present study also increased the odds of bilateral nerve‐sparing (OR 2.49; 95% CI 1.04–5.93, *p* = 0.04) which to our knowledge has not been shown before.

Even though sexual intercourse was possible for 43.1% of patients, 36.5% reported an IIEF5 ≥ 17 at overall follow‐up, and no statistical difference between low‐ and intermediate‐risk patients was found (*p* = 0.646). Slightly higher rates of ED were reported by Haglind et al. [18], who showed IIEF‐5 scores of ≤ 21 (= some ED) in 93% and 90% for ORP and RARP, respectively, using a large cohort of 2625 men. Vickers et al. saw sufficient EF (defined as an IIEF6 score ≥ 22) in 36% of their patients, while Haglind et al. reported ED (defined as lack of stiffness at sexual activity or morning erection) in 70,4% of patients who underwent RARP 12 months after surgery [19].

While the majority of patients reported worse potency after RP, surgical approach (ORP vs. RARP) was associated with improved EF (OR 1.85; 95% CI 1.15–2.99; *p* = 0.012). Our results stand in line with a meta‐analysis by Ficarra et al., who saw a significant benefit for RARP (OR 2.84; 95% CI 1.46–5.43; *p* = 0.002) as well as Haglind et al. (OR 0.81 95% CI, 0.66–0.98) [18, 20]. The high volume of our institution leads to a high experience of the contributing surgeons. Therefore, surgeon volume is a substantial factor for regained EF after ORP/RARP (OR 2.18, 95% CI 1.24–3.83, *p* = 0.006). Overall, different measurements and definitions of EF make an exact evaluation of these above‐mentioned previous studies difficult.

All our findings must be interpreted within their limitations. Our data was collected 10 years ago at a single institution, and especially institution‐specific factors such as a standardized FS template and high surgical volume may not be reproducible at other facilities. No data on the detailed surgical technique of ORP or RARP has been reported. In addition, the utilization of MRI‐guided prostate biopsies is not reflected in this manuscript. Furthermore, the exact extent of NS has not been reported. Patients were not randomized between ORP/RARP, and no current follow‐up is available. Regarding the extrapolation of our results, we acknowledge that no comparison to a control group has been made.

In our cohort, the introduction of whole surface FS expanded the surgeons' ability to perform NS not only to patients with low‐risk PCa, but also to patients with intermediate‐risk PCa. FS increased the number of NS procedures without compromising oncologic safety and provides better functional outcomes for more patients. Therefore, we encourage surgeons to perform surgery cautiously in patients with initially more advanced disease and suggest an individual approach to NS. In contrast to EF, continence was not significantly influenced by more advanced tumor status.

## Author Contributions

**Moritz J. Reike:** conceptualization, investigation, writing – original draft, methodology, writing – review and editing, project administration, software, formal analysis, visualization, validation, data curation. **Karl H. Tully:** software, formal analysis, data curation, supervision, writing – review and editing, funding acquisition, validation. **Maximilian Schulmeier:** conceptualization, data curation, investigation, writing – review and editing. **Alina Reicherz:** writing – review and editing, investigation, validation. **Henning Bahlburg:** investigation, writing – original draft, writing – review and editing. **Guido Müller:** methodology, writing – review and editing, conceptualization, data curation. **Peter Bach:** conceptualization, writing – review and editing, validation, supervision. **Carlo Sternemann:** writing – review and editing, investigation, conceptualization, methodology, validation. **Andrea Tannapfel:** conceptualization, investigation, methodology, validation, writing – review and editing. **Joachim Noldus:** writing – review and editing, conceptualization, supervision. **Marko Brock:** conceptualization, writing – review and editing, supervision. **Florian Roghmann:** supervision, resources, conceptualization, writing – review and editing, writing – original draft.

## Ethics Statement

All procedures performed in studies involving human participants were in accordance with the ethical standards of the institutional and/or national research committee and with the 1964 declaration of Helsinki and its following amendments or comparable ethical standards. The institutional ethics committee approved this study (Approval number 2015‐5250).

## Consent

The institutional ethics committee approved this study (Approval number 2015‐5250) and each patient gave informed consent.

## Conflicts of Interest

Bahlburg H: Speaker honoraria: Desitin; Roghmann F: Speaker honoraria, travel expenses: Janssen, Roche, Merck, MSD, Pfizer, AstraZeneca, QED Advisory Board: Janssen, Roche, Merck, QED.

## Supporting information


Data S1.
