# Supplementary material for: Functional Outcomes in Patients With Prostate Cancer Undergoing Frozen Section Guided Radical Prostatectomy
Source: Int J Urol. 2025 May 20;32(9):1159–64. doi: 10.1111/iju.70115 (PMC12410123; doi:10.1111/iju.70115)
Supplement: Supplementary file 1 — Data S1. [file IJU-32-1159-s001.docx]

**Supplementary files:**

Supplementary figure 1: Standardized template used to report positive surgical margins at the intraoperative frozen section and to guide secondary tumor resection.

**
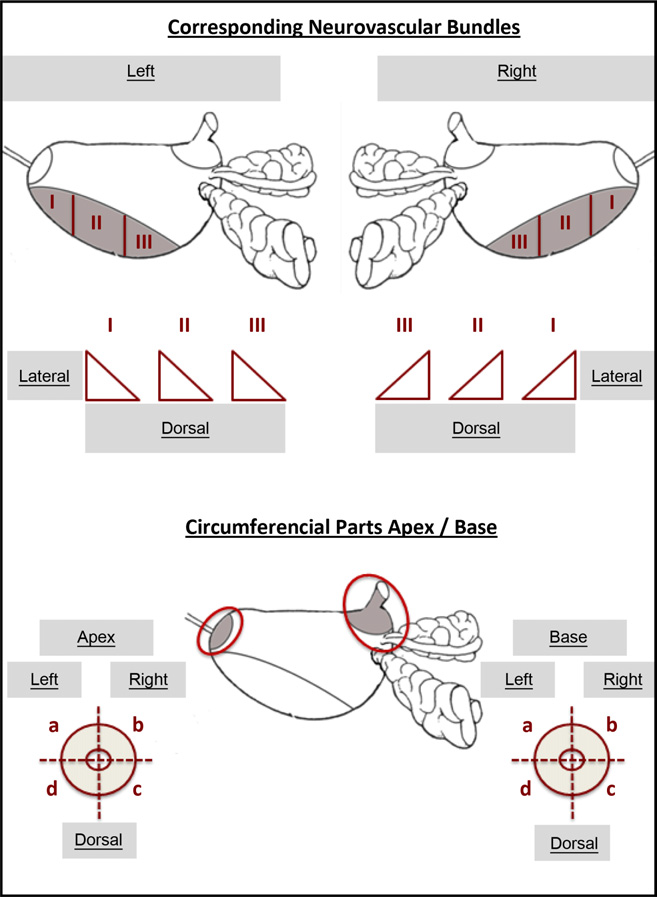
**

| Supplementary Table 1: Multivariable logistic regression analysis examining preoperative predictors of uni- or bilateral nerve sparing at radical prostatectomy | | | | |
| --- | --- | --- | --- | --- |
|  |  | Odds Ratio | 95% CI | p-value |
| Age |  | 0.98 | 0.93 – 1.04 | 0.540 |
| Prostate volume |  | 1.00 | 0.98 – 1.03 | 0.892 |
| Proportion of positive biopsies | < 25 % | 1 [REF] | --- | --- |
|  | 25 – 49 % | 1.01 | 0.35 – 2.95 | 0.982 |
|  | 50 – 74 % | 0.50 | 0.16 – 1.52 | 0.218 |
|  | ≥ 75 % | 0.28 | 0.08 – 1.00 | 0.050 |
| Preoperative PSA | | 0.91 | 0.83 – 1.00 | 0.053 |
| clinical T-stage | cT1 | 1 [REF] | --- | --- |
|  | cT2 | 0.83 | 0.37 – 1.87 | 0.663 |
| Preoperative Gleason Score | ≤6 | 1 [REF] | --- | --- |
|  | 7a | 1.35 | 0.51 – 3.53 | 0.545 |
|  | 7b | 0.39 | 0.15 – 1.01 | 0.053 |
| Surgical approach | Open | 1 [REF] | --- | --- |
|  | Robotic | 0.61 | 0.24 – 1.54 | 0.293 |
| Surgeon volume | Low-Volume | 1 [REF] | --- | --- |
|  | High-Volume | 2.49 | 1.04 – 5.93 | 0.040 |
|  | | | | |

| Supplementary Table 2: Postoperative urinary continence | | | | | |
| --- | --- | --- | --- | --- | --- |
|  | | Overall | Low-risk | Intermediate-/ High-risk | p-value |
| 1-year follow-up (n = 485) | | | | | |
| Median number of pads/24h, n (IQR) | | 0 (0, 1) | 0 (0, 0) | 0 (0, 1) | 0.225 |
| Urinary continence, n (%) | Yes | 402 (82.9) | 143 (84.1) | 259 (82.2) | 0.597 |
|  | No | 83 (17.1) | 27 (15.9) | 56 (17.8) |  |
| Overall follow-up (n = 415) (median follow-up 31 months (IQR 23, 41 months) | | | | | |
| Median number of pads/24h, n (IQR) | | 0 (0, 1) | 0 (0, 1) | 0 (0, 1) | 0.356 |
| Urinary continence, n (%) | Yes | 345 (83.1) | 122 (84.1) | 223 (82.6) | 0.689 |
|  | No | 70 (16.9) | 23 (15.9) | 47 (17.4) |  |
| All p-values were compared to no nerve-sparing. | | | | | |

| Supplementary Table 3: Multivariable logistic regression examining perioperative predictors of urinary continence at 1-year follow-up | | | | |
| --- | --- | --- | --- | --- |
|  |  | Odds Ratio | 95% CI | p-value |
| Age |  | 0.92 | 0.58 – 1.47 | 0.723 |
| Prostate volume |  | 1.00 | 0.99 – 1.02 | 0.653 |
| Preoperative PSA |  | 0.97 | 0.91 – 1.04 | 0.425 |
| Preoperative Gleason Score | 6 | 1 [REF] | --- | --- |
|  | 7a | 2.61 | 1.35 – 5.04 | 0.004 |
|  | 7b | 1.03 | 0.50 – 2.14 | 0.929 |
| Surgical approach | Open | 1 [REF] | --- | --- |
|  | Robotic | 0.84 | 0.45 – 1.57 | 0.584 |
| DRE | cT1 | 1 [REF] | --- | --- |
|  | cT2 | 0.87 | 0.51 – 1.51 | 0.631 |
| Surgeon volume | Low-Volume | 1 [REF] | --- | --- |
|  | High-Volume | 3.85 | 2.19 – 6.75 | <0.001 |
| Nervesparing | None | 1 [REF] | --- | --- |
|  | Unilateral | 1.13 | 0.36 – 3.55 | 0.831 |
|  | Bilateral | 1.33 | 0.43 – 4.08 | 0.621 |
| Urinary continence defined as ≤ 1 pad/24h | | | | |

| Supplementary Table 4: Postoperative functional outcome sexual function in men without prior erectile dysfunction | | | | | |
| --- | --- | --- | --- | --- | --- |
|  | | Overall | Low-risk | Intermediate-/ high-risk | p-value |
| 1-year follow-up | | | | | |
| IIEF 5, n= 137 (%) | <17 | 99 (72.3) | 40 (72.7) | 59 (72.0) | 0.921 |
|  | ≥17 | 38 (27.7) | 15 (27.3) | 23 (28.1) |  |
| Sexual intercourse possible, n=448 (%) | Yes | 155 (34.6) | 64 (42.4) | 91 (30.6) | 0.014 |
|  | No | 293 (65.4) | 87 (57.6) | 206 (69.4) |  |
| Overall follow-up (median follow-up 33 months (IQR 25, 42 months) | | | | | |
| IIEF 5, n = 115 (%) | <17 | 73 (63.5) | 31 (66.0) | 42 (81.8) | 0.646 |
|  | ≥17 | 42 (36.5) | 16 (34.0) | 26 (38.2) |  |
| Sexual intercourse possible, n=404 (%) | Yes | 174 (43.1) | 75 (52.8) | 99 (37.8) | 0.004 |
|  | No | 230 (56.9) | 67 (47.2) | 163 (62.2) |  |

| Supplementary Table 5: Multivariable logistic regression examining perioperative predictors of sufficient erectile function at 1-year follow-up | | | | |
| --- | --- | --- | --- | --- |
|  |  | Odds Ratio | 95% CI | p-value |
| Age |  | 0.91 | 0.88 – 0.95 | <0.001 |
| Prostate volume |  | 0.99 | 0.98 – 1.01 | 0.556 |
| Preoperative PSA | | 0.97 | 0.91 – 1.03 | 0.359 |
| Preoperative Gleason Score | 6 | 1 [REF] | --- | --- |
|  | 7a | 1.15 | 0.69 – 1.90 | 0.587 |
|  | 7b | 0.81 | 0.40 – 1.68 | 0.580 |
| Surgical approach | Open | 1 [REF] | --- | --- |
|  | Robotic | 1.85 | 1.15 – 2.99 | 0.012 |
| DRE | cT1 | 1 [REF] | --- | --- |
|  | cT2 | 0.91 | 0.56 – 1.49 | 0.720 |
| Surgeon volume | Low-Volume | 1 [REF] | --- | --- |
|  | High-Volume | 2.18 | 1.24 – 3.83 | 0.006 |
| Nervesparing | None | 1 [REF] | --- | --- |
|  | Unilateral | 1.12 | 0.30 – 4.22 | 0.865 |
|  | Bilateral | 1.73 | 0.47 – 6.36 | 0.407 |
| Sufficient erectile function defined as IIEF ≥17 or possible sexual intercourse | | | | |
